# Trends in spinal surgery pre- and post-pandemic in a large metropolitan area

**DOI:** 10.1007/s10143-025-03705-9

**Published:** 2025-07-09

**Authors:** Matthew T. Carr, John Caridi

**Affiliations:** 1 Department of Neurosurgery, Icahn School of Medicine, New York, NY USA; 2 Department of Neurosurgery, Lenox Hill Hospital, New York, NY USA

**Keywords:** Ambulatory surgery, COVID-19 pandemic, Demographics, Insurance status, Spine surgery, Volume trends

## Abstract

New York City was hit particularly hard by the COVID-19 pandemic, with lockdowns and elective surgery stoppages. Research into lasting trends in spine surgery volume following the COVID-19 pandemic remains scarce. The primary aim was to identify trends in spine surgery volume and secondary aim was to outline trends in patient characteristics during and after the COVID-19 pandemic in this large urban metropolitan area. The Statewide Planning and Research Cooperative System (SPARCS) database of New York was queried for all adult spine surgery claims from the 5 boroughs of New York City and Westchester County from 2019 to 2023. The principal submitted ICD10 code was used to categorize manually each claim by its associated spinal pathology. There were 26,066 claims in 2019, 20,437 in 2020, 24,829 in 2021, 26,271 claims in 2022, and 30,485 claims in 2023. Total ICD10 diagnoses per claim increased from 9.8 in 2019 to 10.3 in 2023. The proportion of ambulatory claims increased from 36.1% in 2019 to 49.1% in 2023. Length-of-stay averaged 3.23 days in 2019 and fell to 2.80 days by 2023. The percent of patients discharged home rose from 83.5 to 87.0%, with decreases in discharge to skilled nursing facilities (SNF) or rehabilitation. Spine surgery volumes decreased during the pandemic, but in 2022 and 2023 surpassed pre-pandemic numbers. These trends can help guide spine surgeons about ongoing changes in demographics, insurance status, and surgical characteristics, as well as prepare for future pandemics.

**Supplementary Information:**

The online version contains supplementary material available at 10.1007/s10143-025-03705-9.

## Introduction

COVID-19 associated lockdowns and elective surgery stoppages affected spine surgery practices across the United States and the world [13, 22]. The New York City area was hit particularly hard by the pandemic in 2020, with elective surgery restrictions for portions of 2020 and 2021. Other changes to the healthcare landscape during the pandemic included an increase in telehealth services [20, 34], and routine COVID-19 screening before hospital admission or elective procedures. There were also shifts in where people chose to live, business closings, and an increase in remote work that had reverberating effects into healthcare utilization throughout the country.

Much has been published showing decreased spine surgery volumes during peaks of the pandemic [22, 33, 36, 37]. Outcomes may have been worse during the pandemic for those who avoided elective surgery [7, 19], or even those who chose to have surgery during the pandemic regardless [16, 28]. However, research into lasting trends in spine surgery volume following the COVID-19 pandemic remains scarce.

The primary goal of this study is to evaluate trends in spinal surgery volume, both elective and urgent, in the New York City metropolitan area before, during, and after the COVID-19 pandemic. Secondary aims are to evaluate trends in patient demographics, insurance status, and length of stay (LOS) during this period.

## Materials and methods

The New York Department of Health Statewide Planning and Research Cooperative System (SPARCS) database was queried for all adult (age ≥18 years-old) spine surgery claims from the 5 boroughs of New York City and Westchester County from 2019 to 2023. All spinal surgeries, as characterized by Current Procedural Terminology codes, regardless of indication or diagnosis were included. All research was conducted in accordance with the Declaration of Helsinki. This study was deemed exempt from institutional review board approval and the need for informed consent due to the use of deidentified patient data from the SPARCS database. SPARCS is a comprehensive all-payer data collection system established in 1979 as a result of cooperation between the healthcare industry and state government. SPARCS collects patient-level details on patient characteristics, diagnoses and treatments, services, and charges for inpatient and outpatient services [29].

The principal submitted International Classification of Diseases tenth edition (ICD-10) code was used to categorize each claim by its associated primary spinal pathology. A list of ICD-10 codes utilized for each pathology is listed in Supplementary Table 1. Spinal procedures were categorized by their diagnosis related groups (DRG). COVID pandemic affected years are defined as 2020 and 2021 in this study, as these were the years that faced the most substantial barriers to surgical practices via orders to halt surgery as well as resource limitations. Percentages and proportions reported are based on absolute claims numbers from the database. We analyzed the trends in annual number of spinal surgeries using descriptive statistics, one-way ANOVA for continuous variables, and Chi-square test for categorical variables with *p* < 0.05 to indicate significant difference. Analyses were performed using R software, version 4.3.2 (R Foundation for Statistical Computing, Vienna, Austria, 2023). This research did not require institutional review board approval, and all research was conducted in accordance with the Declaration of Helsinki.

## Results

### Surgical volume and diagnoses trends

**Fig. 1:**
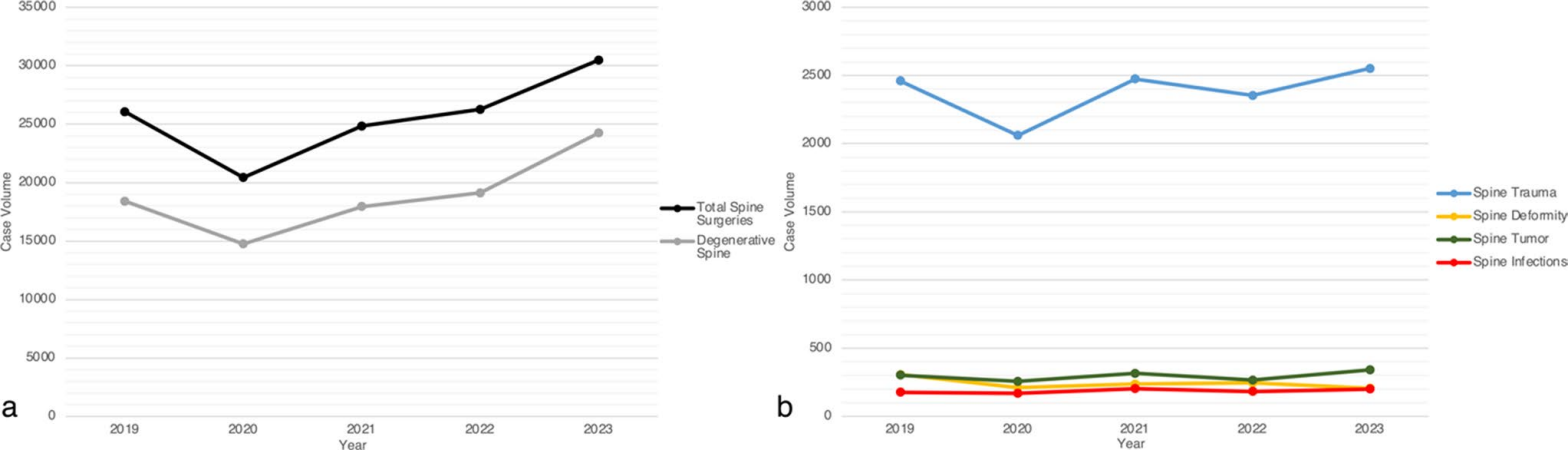
Graph of total spine surgery claims and degenerative spine surgery claims per year (**a**), and annual spine surgery claims for trauma, tumor, deformity, and infection pathologies as determined by principal ICD-10 code (**b**)

There were 26,066 spine surgery claims in 2019, which fell to 20,437 in 2020. Claims rebounded to 24,829 in 2021 before surpassing pre-pandemic levels in 2022 with 26,271 and in 2023 with 30,485 total spinal surgeries. Total ICD10 diagnoses per claim increased from an average of 9.8 in 2019 to 10.4 in 2020, 10.6 in 2021, 10.4 in 2022, and 10.3 in 2023. The percentage of spine trauma surgeries based on primary diagnosis increased from 9.4% of all claims in 2019 to 10.1% in 2020 and 10.0% in 2021, then decreased to 9.0% in 2022 and 8.4% in 2023. The proportion of spine tumor cases rose during the pandemic, with 1.16% in 2019, 1.26% in 2020, and 1.27% in 2021. Spine tumor proportion then decreased post-pandemic with 1.01% in 2022 and 1.12% in 2023. Spine infection cases increased during the pandemic from 0.68% in 2019 to 0.83% in 2020, 0.82% in 2021, and then decreased to 0.70% in 2022 and 0.66% in 2023. Adult spinal deformity decreased from 1.18% in 2019 to 1.03% in 2020, 0.95% in 2021, 0.93% in 2022, and 0.68% in 2023. The majority of claims were for degenerative spine disease, and these claims have been increasing over time with 70.6% in 2019, 72.2% in 2020, 72.3% in 2021, 72.8% in 2022, and 79.5% in 2023. Trends of overall spine surgery volume and proportions of surgery by associated spinal pathology are depicted in Fig. 1; Table 1.

**Table 1:** Overall spine surgery claims and claims by spinal pathology

	2019	2020	2021	2022	2023	*p*-value
Total Spine Surgery claims	26,066	20,437	24,829	26,271	30,485	--
Average ICD-10 diagnoses per claim	9.83	10.41	10.57	10.40	10.31	--
Degenerative Spine claims (%)	18,413 (70.6%)	14,756 (72.2%)	17,953 (72.3%)	19,127 (72.8%)	24,245 (79.5%)	--
Spine Trauma claims (%)	2459 (9.43%)	2060 (10.08%)	2474 (9.96%)	2353 (8.96%)	2552 (8.37%)	--
Spine Deformity claims (%)	307 (1.18%)	211 (1.03%)	237 (0.95%)	245 (0.93%)	206 (0.68%)	--
Spine Tumor claims (%)	302 (1.16%)	257 (1.26%)	315 (1.27%)	265 (1.01%)	341 (1.12%)	--
Spine Infection claims (%)	177 (0.68%)	169 (0.83%)	203 (0.82%)	183 (0.70%)	200 (0.66%)	--
Emergency Surgery claims (%)	8156 (31.29%)	6327 (30.96%)	7339 (29.56%)	7286 (27.73%)	8358 (27.42%)	0.40

### Insurance and ambulatory surgery

The proportion of ambulatory claims has been steadily increasing year over year, from 36.1% of all claims in 2019 to 49.1% in 2023 (*p* < 0.001). The insurance landscape shifted with commercial insurance decreasing from 23.5% in 2019 to 16.5% in 2023. Medicare has also decreased yearly from 36.4% of claims in 2019 down to 31.1% in 2023. Medicaid claims are slightly down post-COVID, from 17.8% in 2019, 17.5% in 2020, 18.2% in 2021, 16.0% in 2022, and 14.7% in 2023. Self-pay/other insurance has increased from 22.2% in 2019 to 37.6% in 2023. The proportion of worker’s compensation claims increased during the period as well, from 6.4% in 2019 to 8.1%, 10.4%, 8.5%, and 8.1% in 2020–2023, respectively although this difference did not reach significance (*p* = 0.32). Insurance and ambulatory status are displayed in Table 2.

**Table 2:** Insurance status and inpatient surgery claims per year

	2019	2020	2021	2022	2023	*p*-value
Inpatient claims (%)	16,647 (63.86%)	12,882 (63.03%)	14,749 (59.40%)	14,213 (54.10%)	15,513 (50.89%)	< 0.001
Commercial/HMO Insurance (%)	6133 (23.53%)	4516 (22.10%)	4857 (19.56%)	4586 (17.46%)	5045 (16.55%)	--
Medicaid (%)	4639 (17.80%)	3578 (17.51%)	4522 (18.21%)	4212 (16.03%)	4491 (14.73%)	--
Medicare (%)	9507 (36.47%)	6987 (34.19%)	8142 (32.79%)	8239 (31.36%)	9480 (31.10%)	--
Self Pay/ Other (%)	5787 (22.20%)	5356 (26.21%)	7308 (29.43%)	9234 (35.15%)	11,469 (37.62%)	--
Worker’s Compensation claim (%)	1680 (6.45%)	1651 (8.08%)	2589 (10.43%)	2230 (8.49%)	2465 (8.09%)	0.32

### Patient demographics

The average age of patients has been steadily decreasing over this period, from 56.6 years old in 2019 down to 55.8 years old in 2020, 55.3 years old in 2021, 54.7 years old in 2022, and 54.1 years old in 2023 (*p* < 0.001). The race and ethnicity breakdown of patients in the New York City metropolitan area has also been changing. The proportion of White patients has decreased from 34.2% in 2019 to 26.5% in 2023. The percentage of Black and Hispanic patients are mildly decreased, from 19.3% in 2019 to 15.3% in 2023 for patients identifying as Black/African American and from 21.0% in 2019 to 19.3% in 2023 for those identifying as Hispanic/Latino. Those identifying as Asian or other race have been slowly increasing. Asian patients remained stable from 5.4% in 2019 to 5.1% in 2023. Other race has increased from 19.2% in 2019 to 32.6% in 2023 (*p* < 0.001). Table 3 lists age and race statistics in greater detail.

**Table 3:** Age, race, insurance status, and inpatient status of spine surgery patients by year

	2019	2020	2021	2022	2023	*p*-value
Average Age	56.60	55.77	55.26	54.67	54.13	< 0.001
American Indian/Alaskan Native (%)	48 (0.18%)	28 (0.14%)	25 (0.10%)	39 (0.15%)	67 (0.22%)	< 0.001
Asian (%)	1399 (5.37%)	1034 (5.06%)	1385 (5.58%)	1550 (5.90%)	1557 (5.11%)	
Black/ African American (%)	5042 (19.34%)	3793 (18.56%)	4857 (19.56%)	4816 (18.33%)	4673 (15.33%)	
Hispanic/Latino (%)	5487 (21.05%)	3866 (18.92%)	5179 (20.86%)	5364 (20.42%)	5881 (19.29%)	
Multi Racial (%)	162 (0.62%)	197 (0.96%)	182 (0.73%)	252 (0.96%)	282 (0.93%)	
Native Hawaiian or Pacific Islander (%)	23 (0.09%)	26 (0.13%)	15 (0.06%)	17 (0.06%)	25 (0.08%)	
Other Race (%)	5003 (19.19%)	4951 (24.23%)	5819 (23.44%)	6972 (26.54%)	9935 (32.59%)	
White (%)	8902 (34.15%)	6542 (32.01%)	7367 (29.67%)	7261 (27.64%)	8065 (26.46%)	

### Length of stay and discharge destination

Average hospital LOS has also been decreasing over this time, in keeping with the increasing proportion of ambulatory surgeries. Including ambulatory patients, LOS averaged 3.23 days in 2019 and has shortened to 3.15, 3.06, 2.97, and 2.80 days over the subsequent 4 years (*p* < 0.001). However, when accounting only for inpatient surgeries, LOS was roughly stable from 2019 at 5.06 days to 2021 at 5.15 days, further increasing post-pandemic to 5.49 days in 2022 and 5.50 days in 2023. The percentage of patients discharged to home has steadily risen during and after the pandemic. 83.5% of patients were discharged to home in 2019, increasing to 84.8%, 85.3%, 86.1%, and 87.0% each year thereafter. Discharges to inpatient rehabilitation and skilled nursing facilities (SNF) both decreased over time from 2019 to 2022. Discharge to rehabilitation changed from 4.4% in 2019 to 3.3% in 2023. Similarly, discharge to SNF fell from 9.8% in 2019 to 7.6% in 2023. Patients who expired or were discharged to hospice care increased during the pandemic, from 0.30% in 2019 up to 0.46% in both 2020 and 2021, and then decreased to 0.37% in 2022 and 0.29% in 2023. Table 4 lists all discharge destinations.

**Table 4:** Discharge destinations and length of stay following spine surgery

	2019	2020	2021	2022	2023	*p*-value
Average LOS for all patients (days)	3.23	3.15	3.06	2.97	2.80	< 0.001
Average LOS for inpatients (days)	5.06	5.00	5.15	5.49	5.50	--
Home (%)	21,786 (83.58%)	17,337 (84.83%)	21,186 (85.33%)	22,624 (86.12%)	26,514 (86.97%)	--
Inpatient Rehabilitation (%)	1141 (4.38%)	897 (4.39%)	1056 (4.25%)	999 (3.80%)	1013 (3.32%)	--
Skilled Nursing Facility (%)	2548 (9.78%)	1689 (8.26%)	1962 (7.90%)	2058 (8.00%)	2330 (7.64%)	--
Expired/Hospice (%)	78 (0.30%)	94 (0.46%)	114 (0.46%)	96 (0.37%)	89 (0.29%)	--
Other (%)	513 (1.97%)	420 (2.06%)	511 (2.06%)	494 (1.88%)	539 (1.77%)	--

LOS = length of stay

### Surgical characteristics

Of the spine surgery claims in 2019, 8,156 (31.3%) were listed as “emergency” surgeries. The proportion of emergency surgeries has steadily fallen every year since, with 6,327 (30.9%) in 2020, 7,339 (29.6%) in 2021, 7,286 (27.7%) in 2022, and 8,358 (27.4%) in 2023, however this difference was not significant (*p* = 0.40). The proportion of each inpatient DRG group has been decreasing over time due to the steadily increasing number of ambulatory surgeries, which carries its own distinct DRG in the dataset. When comparing only inpatient DRGs, however, different patterns emerge. “Combined anterior/posterior spinal fusion” (DRGs 453–455) have steadily been increasing as a percentage of inpatient spinal surgery DRGs from 12.8% in 2019 to 13.8% in 2023. “Medical back problems” (DRGs 551–552) decreased during COVID years, from 37.3% of all inpatient DRGs in 2019 down to 35.7% and 36.6% in 2020 and 2021, then increased above the pre-pandemic numbers to 38.2% in 2022 and 41.6% in 2023. The proportion of spinal fusion DRGs (including DRGs 453–460 and 471–473) increased during COVID from 46.7% of all inpatient DRGs in 2019 to 47.8% and 47.7% in 2020–2021, before falling in 2022 to 45.6% and in 2023 to 42.8%. Table 5 summarizes the frequency of DRG codes per year, and Supplementary Table 2 includes a more detailed breakdown of DRG codes.

**Table 5:** Diagnosis related groups per year as a percentage of claims

DRG Codes	DRG Description	2019	2020	2021	2022	2023
Amb Surg	Ambulatory Surgery (% of total claims)	9419 (36.14%)	7555 (36.97%)	10,080 (40.60%)	12,058 (45.90%)	14,972 (49.11%)
028–030	Spinal Procedures or Spinal Neurostimulators (% of inpatient claims)	666 (4.0%)	513 (4.0%)	569 (3.9%)	608 (4.3%)	624 (4.0%)
052–053	Spinal Disorders & Injuries (% of inpatient claims)	251 (1.5%)	221 (1.7%)	192 (1.3%)	218 (1.5%)	206 (1.3%)
453–455	Combined Anterior/Posterior Spinal Fusion (% of inpatient claims)	2124 (12.8%)	1729 (13.4%)	2071 (14.0%)	2103 (14.8%)	2141 (13.8%)
456–458	Spinal Fusion Except Cervical with Spinal Curvature or Malignancy or Infection or Extensive Fusions (% of inpatient claims)	435 (2.6%)	337 (2.6%)	421 (2.9%)	361 (2.5%)	450 (2.9%)
459–460	Spinal Fusion Except Cervical (% of inpatient claims)	2512 (15.1%)	1934 (15.0%)	2112 (14.3%)	1828 (12.9%)	1729 (11.1%)
471–473	Cervical Spinal Fusion (% of inpatient claims)	2707 (16.3%)	2164 (16.8%)	2433 (16.5%)	2183 (15.4%)	2319 (14.9%)
518–520	Back & Neck Procedures Except Spinal Fusion (% of inpatient claims)	1744 (10.5%)	1390 (10.8%)	1558 (10.6%)	1487 (10.5%)	1589 (10.2%)
551–552	Medical Back Problems (% of inpatient claims)	6208 (37.3%)	4594 (35.7%)	5393 (36.6%)	5425 (38.2%)	6455 (41.6%)

DRG = diagnosis related group

## Discussion

The COVID-19 pandemic greatly affected spine surgery volumes during the associated lockdowns and initial waves worldwide, as has been shown before [5, 9, 13, 18, 22, 27, 33]. This study is among the first to show that even in a major metropolitan area like New York City that was the epicenter of the pandemic nationally, by 2022 and 2023 spinal surgery volume rebounded above pre-pandemic levels. Spinal surgery in the United States has been on an increasing trajectory, with predictions that these cervical and lumbar fusions will continue to grow through 2040 [24, 25]. The pandemic thus may have only temporarily bucked that trend, and spinal surgeries have now continued to increase as predicted. The rebound over pre-pandemic levels could also reflect pent-up demand from surgeries delayed during the pandemic.

Of the spine surgery principal diagnoses we examined, there was a slight increase in the percentage of spine trauma, tumor, and infection diagnoses during the COVID years of 2020 and 2021, with a progressive decrease in adult spinal deformity diagnoses. It is logical to suspect that surgeries for pathologies such as trauma, tumor, and infection, which may present as more urgent/emergent cases than many degenerative diseases, may increase in relative frequency during the pandemic when elective surgeries were stopped or limited. However, none of these increases were substantial. Given the relative stability of the raw number of spine tumor and infection surgeries year-by-year, any proportional change in tumor or infection cases could be related to decreases in elective surgeries during the pandemic and increase in elective surgeries after the pandemic resulting in proportional increase or decrease of spine tumor/infection cases. Degenerative spine percentage increased year over year in this sample; that trend most closely mirrors the trend of overall spine surgeries during the timeframe, which is logical given most surgeries are for degenerative conditions. Previous research into the presentation and volumes of particular spinal pathologies during COVID is scarce and conflicting. A study in the United Kingdom found that referrals for spine tumors, high energy trauma, and infections were all decreased during the pandemic [2]. A national database study by Tarawneh et al. found that most spinal procedures and diagnoses were decreased during the pandemic except for open reduction of thoracic fractures [33]. In Nepal, overall spinal surgery volumes were decreased but the proportion of surgeries performed for spine trauma increased during COVID [9]. For spinal tumors, a study by Mika et al. found that fewer patients underwent surgery for spinal metastases during COVID and those who had surgery had higher spinal instability neoplastic scores (SINS) and lower Tokuhashi scores [21]. A study examining spinal fusion for spinal infections in Germany found no significant change in number of surgeries for infection during the COVID waves [1]. In spinal deformity, elective adolescent idiopathic scoliosis surgery delays were found to lead to worse clinical and radiographic outcomes [7].

Oddly, the relative frequency of surgeries categorized as “emergent” decreased during and after the pandemic compared to pre-pandemic levels. Lockdowns or migration outside the city during the pandemic could partially explain the decreased number of emergent surgeries, at least during the height of the pandemic. A similar decrease in emergency surgeries for spinal cord injury was noted in Australia during the pandemic [26]. However, other studies have found increased proportions of “urgent” spine surgeries during the pandemic [4, 30].

This study also found an increase in percentage of inpatient surgeries involving fusion, as determined by DRG, during the COVID years. Combined anterior/posterior fusions have been steadily increasing as a proportion of all inpatient surgeries every year, even after COVID. The reason for these patterns is unclear. Patients and surgeons may have avoided potentially less-urgent surgeries requiring decompression only during the pandemic. The trend of increasing spinal fusions overall has been noted previously [17, 24, 25].

The increase in percentage of inpatient spinal fusions could also reflect the annually increasing number of ambulatory spine surgeries performed during this period. Outpatient and ambulatory spine surgery is becoming more common [11, 15]. This is due to a multitude of factors outside of the COVID pandemic including the need to reduce healthcare expenditures and costs, as well as advances in minimally-invasive surgery making outpatient spine surgery more feasible [8, 11]. The pandemic may have driven this shift to ambulatory surgery through patient and surgeon preferences to avoid hospitalization. A recent study by Cassimatis et al. found a slight patient preference for surgery in ambulatory surgery center versus a hospital during COVID, especially among patients more afraid of contracting the virus [6]. However, more complex surgeries requiring hospitalization remain inpatient surgeries, and it may be that a larger portion of these are fusions. The number of comorbid ICD-10 diagnoses per patient increased during and after COVID, from 9.83 ICD-10 codes per claim in 2019 to 10.31 in 2023, which may reflect that more sick/complex patients were undergoing spine surgery during and after the pandemic. Or this increase could be due to COVID itself or the lasting effects of the virus adding a diagnosis to many patients after the initial outbreak. A national database study similarly found an increase in number of comorbidities of patients undergoing cervical surgery during COVID [16]. The increasing medical complexity of spine patients, regardless of the cause, should be noted by spine surgeons and may require additional medical work-up and optimization prior to spinal surgery.

Hospital LOS decreased every year in this study for all surgeries, but LOS slightly increased when looking at inpatient surgeries alone. Prior studies have shown shorter hospital LOS during COVID [4, 35]. A pre-pandemic evaluation of neurosurgical procedure trends using the National Inpatient Sample found that for all DRGs, including spinal fusions, LOS was decreasing or staying the same [17]. The percentage of home discharges following spine surgery also increased during COVID and in 2022, with fewer patients each year being discharged to SNF or acute rehabilitation. The increase in home discharges and decrease in SNF discharges could reflect broader post-acute care trends. The need to cut healthcare costs and/or lack of availability of SNF beds may result in increased availability of post-surgical home health services, including nursing care and therapy, allowing more spine surgery patients to recover at home. The trend of more home discharges following adult spinal deformity surgeries during COVID has been noted previously [35]. A slight increase in in-hospital mortality and hospice discharges during COVID is understandable given the initial high mortality rates of hospitalized COVID patients, especially during the initial outbreak in New York. However, a study after the resumption of elective surgeries after the first wave in the United Kingdom found that no spine surgery patients developed COVID or died from COVID [23], so it is possible that a factor outside of COVID was responsible for the increase in mortality rates after 2019.

The demographics of spine surgery patients changed over this time. The age of patients gradually decreased every year. This differs from a prior study of neurosurgical trends in which the average age for many spinal fusion DRGs was increasing or statistically unchanged over time [17]. The decrease in age could reflect overall population health or lifestyle changes that are resulting in younger patients developing spinal pathology. Or, the decreasing age may be confounded by other variables such as shifts in insurance coverage or surgical indications. Most drastically, the percentage of White patients substantially decreased, and the percentage of “Other” race patients increased in the study period. There were smaller changes in percentage of Black, Hispanic/Latino, and Asian patients. These racial changes may reflect the overall changing demographics of the metropolitan area at large. However, the decrease in White patients and corresponding increase in Hispanic, Black, and Asian patients were also noted in two recent reviews of nationwide cervical and lumbar fusion data [36, 37]. The shift in patient race demographics may be a result of greater access to spine surgery care for racial minority patients, possibly via changes in patient insurance status or more surgeon availability in previously-underserved areas. Given the noted racial disparities in spine surgery access and outcomes, it is important for spinal surgeons to be cognizant of the changing demographics and provide equitable care [3, 10, 12]. A recent systematic review by Akosman et al. found that African American patients were more likely than White patients to experience readmission, non-routine discharge, medical complications, surgical complications, and mortality following elective spine surgery [3]. Thus, while improving access to spine surgery for racial minorities is important, it is as important that non-White patients receive the same safe and low-complication spine care as White patients.

The health insurance landscape for spine surgery patients has also changed substantially. Commercially insured patients decreased the most, with Medicare and Medicaid also decreasing while self-pay/other has greatly increased. The shift in insurance type could reflect economic/labor changes due to the pandemic. The number of Workman’s comp cases has also increased in the area. Patient insurance status has implications on physician reimbursement for spine surgery, as well as patient outcomes. Medicaid, self-pay/other, and workman’s compensation have all been linked to worse outcomes following common spinal surgeries [14, 31, 32]. Knowledge of shifting insurance/payor status could also guide future surgical and hospital resource planning.

### Limitations

This is a descriptive, retrospective database study and is subject to the limitations inherent to these studies. The lack of specific dates of surgery/ admission did not allow us to narrow down our analysis beyond the year of admission. This data comes from the New York City metropolitan area, and while this area was particularly affected by the peak of the pandemic, the trends seen here may lack generalizability to other portions of the country or the world. The claim-level dataset available lacked more granular data regarding costs, or specifics of surgery including which claims involved minimally-invasive procedures, surgical approach, or number of levels. The lack of granularity also limits our analysis. There were no missing data points in the dataset utilized, and it is possible that claims with missing data were not included in the dataset, or which could introduce bias. Coding errors in the dataset are distinctly possible and would also lead to inaccurate analyses. DRGs were provided in the dataset, but coding of DRGs could vary significantly between healthcare systems which would affect these analyses. The categorization of claims by ICD-10 code only accounted for principal ICD-10 codes submitted, and any potentially ambiguous or nonspecific principal ICD-10 codes were not assigned to one of the subgroups. For these reasons, it is possible that the analyzed spinal pathologies were underestimated, and that assigning different ICD codes to these categories could show different results.

## Conclusions

Retrospective analysis of a state insurance database of spinal surgery claims from 2019 to 2023 reveals that in the New York City metropolitan area overall spine surgery volume fell during the pandemic years of 2020 and 2021, but in 2022 and 2023 rebounded above pre-pandemic amounts. Health systems in the region demonstrated great resilience in rebuilding spine surgery volume. Ambulatory surgeries increased annually while average LOS across all surgeries decreased annually during this period. LOS for only inpatient surgeries increased slightly from 2019 to 2023. Patient insurance shifted from less commercial insurance and Medicare over this period to more self-pay/other insurance. The pandemic and associated recovery may have altered patient demographics, comorbidities, and insurance status, which can guide spine surgery planning. Future prospective longitudinal studies monitoring post-pandemic recovery are required to determine how robust these trends are.

## Electronic supplementary material

Below is the link to the electronic supplementary material.

Supplementary Material 1

Supplementary Material 2

## Data Availability

No datasets were generated or analysed during the current study.

## Declarations

### SPARCS data disclaimer

This publication was produced from raw data provided by the New York State Department of Health (NYSDOH). However, the conclusions derived, calculations, and views expressed herein are those of the authors and do not reflect the conclusions or views of NYSDOH. NYSDOH, its employees, officers, and agents make no representation, warranty or guarantee as to the accuracy, completeness, currency, or suitability of the information provided here.
