# Supplementary material for: Trends in spinal surgery pre- and post-pandemic in a large metropolitan area
Source: Neurosurg Rev. 2025 Jul 9;48(1):549. doi: 10.1007/s10143-025-03705-9 (PMC12238211; doi:10.1007/s10143-025-03705-9)
Supplement: Supplementary file 1 — Supplementary Material 1 [file 10143_2025_3705_MOESM1_ESM.docx]

Supplementary Table 1: ICD-10 diagnoses used to categorize spine surgery claims by associated pathology

| **ICD-10 Trauma codes** | **Code Description** | **ICD-10 Deformity Codes** | **Code Description** | **ICD-10 Tumor Codes** | **Code Description** | **ICD-10 Infection codes** | **Code Description** | **ICD-10 Degenerative Codes** | **Code Description** |
| --- | --- | --- | --- | --- | --- | --- | --- | --- | --- |
| G82 | Paraplegia and tetraplegia | M40 | Kyphosis | C41.2 | Malignant neoplasm of vertebral column | A17 | Tuberculous meningitis | M43.0 | Spondylolysis |
| S12.0 | Fracture of first cervical vertebra | M41 | Idiopathic scoliosis | C41.4 | Malignant neoplasm of sacrum | A18.0 | Tuberculosis of bone | M43.1 | Spondylolisthesis |
| S12.1 | Unspecified displaced fracture of second cervical vertebra, initial encounter for closed fracture | M42.0 | Juvenile osteochondrosis of spine, site unspecified | C70.1 | Malignant Neoplasm of spine meninges | G06.1 | Intraspinal abscess | M45.9 | Ankylosing spondylitis of unspecified sites in spine |
| S12.2 | Fracture of other specified cervical vertebra | Q67.5 | Congenital deformity of spine | C70.9 | Malignant Neoplasm of unspecified meninges | G06.2 | Nervous abscess, unspecified | M47.1 | Other spondylosis with myelopathy |
| S12.3 | Unspecified displaced fracture of fourth cervical vertebra, initial encounter for closed fracture | Q76.3 | Congenital scoliosis due to congenital bony malformation | C72.0 | Malignant neoplasm of spinal cord | G07 | Nervous system abscess/granuloma | M47.2 | Other spondylosis with radiculopathy |
| S12.4 | Fracture of fifth cervical vertebra |  |  | C72.1 | Malignant neoplasm of cauda equina | M46.1 | Sacroiliitis, not elsewhere classified | M47.8 | Other spondylosis |
| S12.9 | Fracture of neck, unspecified, initial encounter |  |  | C72.8 | Malignant neoplasm overlapping | M46.2 | Osteomyelitis of vertebra, site unspecified | M47.9 | Spondylosis, unspecified |
| S13.0 | Traumatic rupture of cervical intervertebral disk |  |  | C72.9 | Malignant neoplasm unspecified | M46.3 | Infection of intervertebral disc (pyogenic) | M48.0 | Spinal stenosis |
| S13.1 | Dislocation of unspecified cervical vertebrae, initial encounter |  |  | C79.4 | Secondary malignant neoplasm of other parts of nervous system | M46.4 | Discitis, unspecified, thoracolumbar | M48.1 | Ankylosing hyperostosis [Forestier] |
| S13.2 | Dislocation of other and unspecified parts of neck |  |  | C79.5 | secondary malignant neoplasm of bone and bone marrow | M46.5 | Other infective spondylopathies | M48.9 | Spondylopathy, unspecified |
| S13.4 | Sprain and strain of cervical spine |  |  | C80 | Unknown primary | M49.0 | Pott disease | M50 | Cervical disc disorders unspecified cervical region |
| S14.0 | Concussion and edema of cervical spinal cord |  |  | D16.6 | benign neoplasm of vertebral column bone | M49.1 | Brucellosis of spine | M51 | Thoracic, thoracolumbar, and lumbosacral intervertebral disc disorders |
| S14.1 | Other and unspecified injuries of cervical spinal cord |  |  | D16.8 | Benign neoplasm of sacral bone | M49.2 | enterobacterial spondylitis | M54.1 | Radiculopathy |
| S22.0 | Fracture of thoracic vertebra |  |  | D18.02 | Hemangioma of intracranial structures and any site | M49.3 | spondylopathy in other infection | M54.2 | Cervicalgia |
| S23.1 | Dislocation of thoracic vertebra |  |  | D32 | Benign neoplasm of meninges (cerebral, spinal and unspecified) | T81.4 | Infection following a procedure | M54.3 | Sciatica |
| S24.0 | Concussion and edema of thoracic spinal cord |  |  | D33 | Benign neoplasm of brain and other parts of central nervous system | T84.7 | infection of implanted orthopedic device | M54.4 | Lumbago with Sciatica |
| S24.1 | Other and unspecified injuries of thoracic spinal cord |  |  | D42 | Neoplasm of uncertain or unknown behavior of meninges, brain, CNS | T85.7 | infection of other implanted device | M54.5 | Low back pain |
| S32.0 | Fracture of lumbar vertebra |  |  | D43 | Neoplasm of uncertain or unknown behavior of meninges, brain, CNS |  |  | M54.6 | Pain in thoracic spine |
| S32.1 | Unspecified fracture of sacrum, initial |  |  | D49.6 | Neoplasm of unspecified behavior of brain, endocrine glands and other CNS |  |  | M54.8 | Other dorsalgia |
| S32.2 | Fracture of coccyx, initial encounter for closed fracture |  |  | D49.7 | Neoplasm of unspecified behavior of brain, endocrine glands and other CNS |  |  | M54.9 | dorsalgia, unspecified |
| S33.1 | Dislocation of lumbar vertebra |  |  | M84.4 | Pathologic fracture |  |  | Q76.2 | Congenital spondylolisthesis |
| S34.0 | Concussion and edema of lumbar spinal cord |  |  | M85.4 | Bone Cyst |  |  |  |  |
| S34.1 | Other injury of lumbar spinal cord |  |  | M85.5 | ABC |  |  |  |  |
| S34.3 | Injury of cauda equina |  |  | M85.6 | Bone Cyst |  |  |  |  |
| T06.0 | Injuries of brain and cranial nerves with injuries of nerves and spinal cord at neck level |  |  | Q85.0 | Neurofibromatosis (nonmalignant) |  |  |  |  |
| T06.1 | Injuries of nerves and spinal cord involving other multiple body regions |  |  |  |  |  |  |  |  |
| T09.3 | Injury of spinal cord, level unspecified |  |  |  |  |  |  |  |  |
| T91.1 | Sequelae of injuries, of poisoning and of other consequences of external causes—Sequelae of injuries of neck and trunk—Sequelae of fracture of spine |  |  |  |  |  |  |  |  |
| T91.3 | Sequelae of injuries, of poisoning and of other consequences of external causes—Sequelae of injuries of neck and trunk—Sequelae of injury of spinal cord |  |  |  |  |  |  |  |  |
