# Supplementary material for: Trends in spinal surgery pre- and post-pandemic in a large metropolitan area
Source: Neurosurg Rev. 2025 Jul 9;48(1):549. doi: 10.1007/s10143-025-03705-9 (PMC12238211; doi:10.1007/s10143-025-03705-9)
Supplement: Supplementary file 2 — Supplementary Material 2 [file 10143_2025_3705_MOESM2_ESM.docx]

Supplementary Table 2: Diagnosis Related Groups per Year as a Percentage of Total Claims and Inpatient-only Claims

| **DRG Number** | **DRG Description** | **2019** | **2020** | **2021** | **2022** | **2023** |
| --- | --- | --- | --- | --- | --- | --- |
| 028 | Spinal Procedures W Mcc (% of total claims) | 150 (0.58%) | 142 (0.69%) | 148 (0.60%) | 167 (0.64%) | 156 (0.51%) |
|  | % of inpatient claims | 0.90% | 1.10% | 1.00% | 1.17% | 1.00% |
| 029 | Spinal Procedures W Cc Or Spinal Neurostimulators (% of total claims) | 342 (1.31%) | 260 (1.27%) | 303 (1.22%) | 293 (1.12%) | 322 (1.06%) |
|  | % of inpatient claims | 2.05% | 2.02% | 2.05% | 2.06% | 2.08% |
| 030 | Spinal Procedures W/O Cc/Mcc (% of total claims) | 174 (0.67%) | 111 (0.54%) | 118 (0.48%) | 148 (0.56%) | 146 (0.48%) |
|  | % of inpatient claims | 1.05% | 0.86% | 0.80% | 1.04% | 0.94% |
| 052 | Spinal Disorders & Injuries W Cc/Mcc (% of total claims) | 211 (0.81%) | 185 (0.91%) | 157 (0.63%) | 185 (0.70%) | 178 (0.58%) |
|  | % of inpatient claims | 1.27% | 1.44% | 1.06% | 1.30% | 1.15% |
| 053 | Spinal Disorders & Injuries W/O Cc/Mcc (% of total claims) | 40 (0.15%) | 36 (0.18%) | 35 (0.14%) | 33 (0.13%) | 28 (0.09%) |
|  | % of inpatient claims | 0.24% | 0.28% | 0.24% | 0.23% | 0.18% |
| 453 | Combined Anterior/Posterior Spinal Fusion W Mcc (% of total claims) | 164 (0.63%) | 135 (0.66%) | 192 (0.77%) | 214 (0.81%) | 228 (0.75%) |
|  | % of inpatient claims | 0.99% | 1.05% | 1.30% | 1.51% | 1.47% |
| 454 | Combined Anterior/Posterior Spinal Fusion W Cc (% of total claims) | 987 (3.79%) | 865 (4.23%) | 1055 (4.25%) | 1067 (4.06%) | 1055 (3.46%) |
|  | % of inpatient claims | 5.93% | 6.71% | 7.15% | 7.51% | 6.80% |
| 455 | Combined Anterior/Posterior Spinal Fusion W/O Cc/Mcc (% of total claims) | 973 (3.73%) | 729 (3.57%) | 824 (3.32%) | 822 (3.13%) | 858 (2.81%) |
|  | % of inpatient claims | 5.84% | 5.66% | 5.59% | 5.78% | 5.53% |
| 456 | Spinal Fusion Except Cervical with Spinal Curvature or Malignancy or Infection or Extensive Fusions W Mcc (% of total claims) | 108 (0.41%) | 100 (0.49%) | 122 (0.49%) | 124 (0.47%) | 110 (0.36%) |
|  | % of inpatient claims | 0.65% | 0.78% | 0.83% | 0.87% | 0.71% |
| 457 | Spinal Fusion Except Cervical with Spinal Curvature or Malignancy or Infection or Extensive Fusions W Cc (% of total claims) | 267 (1.02%) | 193 (0.94%) | 240 (0.97%) | 183 (0.70%) | 262 (0.86%) |
|  | % of inpatient claims | 1.60% | 1.50% | 1.63% | 1.29% | 1.69% |
| 458 | Spinal Fusion Except Cervical with Spinal Curvature or Malignancy or Infection or Extensive Fusions W/O Cc/Mcc (% of total claims) | 60 (0.23%) | 44 (0.22%) | 59 (0.24%) | 54 (0.21%) | 78 (0.26%) |
|  | % of inpatient claims | 0.36% | 0.34% | 0.40% | 0.38% | 0.50% |
| 459 | Spinal Fusion Except Cervical W Mcc (% of total claims) | 191 (0.73%) | 127 (0.62%) | 176 (0.71%) | 156 (0.59%) | 182 (0.60%) |
|  | % of inpatient claims | 1.15% | 0.99% | 1.19% | 1.10% | 1.17% |
| 460 | Spinal Fusion Except Cervical W/O Mcc (% of total claims) | 2321 (8.90%) | 1807 (8.84%) | 1936 (7.80%) | 1672 (6.36%) | 1547 (5.07%) |
|  | % of inpatient claims | 13.94% | 14.03% | 13.13% | 11.76% | 9.97% |
| 471 | Cervical Spinal Fusion W Mcc (% of total claims) | 181 (0.69%) | 165 (0.81%) | 182 (0.73%) | 196 (0.75%) | 189 (0.62%) |
|  | % of inpatient claims | 1.09% | 1.28% | 1.23% | 1.38% | 1.21% |
| 472 | Cervical Spinal Fusion W Cc (% of total claims) | 1257 (4.82%) | 1067 (5.22%) | 1148 (4.62%) | 1048 (3.99%) | 1215 (3.99%) |
|  | % of inpatient claims | 7.55% | 8.28% | 7.78% | 7.37% | 7.83% |
| 473 | Cervical Spinal Fusion W/O Cc/Mcc (% of total claims) | 1269 (4.87%) | 932 (4.56%) | 1103 (4.44%) | 939 (3.57%) | 915 (3.00%) |
|  | % of inpatient claims | 7.62% | 7.23% | 7.48% | 6.61% | 5.90% |
| 518 | Back & Neck Procedures Except Spinal Fusion W Mcc Or Disc Device/Neurostimulator (% of total claims) | 216 (0.83%) | 187 (0.92%) | 202 (0.81%) | 219 (0.83%) | 242 (0.79%) |
|  | % of inpatient claims | 1.30% | 1.45% | 1.37% | 1.54% | 1.56% |
| 519 | Back & Neck Procedures Except Spinal Fusion W CC (% of total claims) | 694 (2.66%) | 585 (2.86%) | 679 (2.73%) | 616 (2.34%) | 690 (2.26%) |
|  | % of inpatient claims | 4.17% | 4.54% | 4.60% | 4.33% | 4.45% |
| 520 | Back & Neck Procedures Except Spinal Fusion W/O Cc/Mcc (% of total claims) | 834 (3.20%) | 618 (3.02%) | 677 (2.73%) | 652 (2.48%) | 657 (2.16%) |
|  | % of inpatient claims | 5.01% | 4.80% | 4.59% | 4.59% | 4.24% |
| 551 | Medical Back Problems W Mcc (% of total claims) | 863 (3.31%) | 730 (3.57%) | 848 (3.42%) | 1010 (3.84%) | 1029 (3.38%) |
|  | % of inpatient claims | 5.18% | 5.67% | 5.75% | 7.11% | 6.63% |
| 552 | Medical Back Problems W/O Mcc (% of total claims) | 5345 (20.51%) | 3864 (18.91%) | 4545 (18.31%) | 4415 (16.81%) | 5426 (17.80%) |
|  | % of inpatient claims | 32.11% | 30.00% | 30.82% | 31.06% | 34.98% |

DRG= diagnosis related group
